# What evidence is there to support skill mix changes between GPs, pharmacists and practice nurses in the care of elderly people living in the community?

**DOI:** 10.1186/1743-8462-6-23

**Published:** 2009-09-11

**Authors:** Sarah Dennis, Jenny May, David Perkins, Nicholas Zwar, Bonnie Sibbald, Iqbal Hasan

**Affiliations:** 1 Centre for Primary Health Care and Equity, School of Public Health and Community Medicine, University of New South Wales, Sydney, NSW, 2052, Australia; 2 University Department of Rural Health (UDRH), University of Newcastle, Locked Bag 9783, New England Mail Sorting Centre, NSW, 2348, Australia; 3 Broken Hill University Department of Rural Health, University of Sydney, PO Box 457, Broken Hill, NSW, 2880, Australia; 4 School of Public Health and Community Medicine, University of New South Wales, Sydney, NSW, 2052, Australia; 5 National Primary Care Research and Development Centre (NPCRDC), University of Manchester, Williamson Building, Oxford Road, Manchester, M13 9PL, UK

## Abstract

**Background:**

Workforce shortages in Australia are occurring across a range of health disciplines but are most acute in general practice. Skill mix change such as task substitution is one solution to workforce shortages. The aim of this systematic review was to explore the evidence for the effectiveness of task substitution between GPs and pharmacists and GPs and nurses for the care of older people with chronic disease. Published, peer reviewed (black) and non-peer reviewed (grey) literature were included in the review if they met the inclusion criteria.

**Results:**

Forty-six articles were included in the review. Task substitution between pharmacists and GPs and nurses and GPs resulted in an improved process of care and patient outcomes, such as improved disease control. The interventions were either health promotion or disease management according to guidelines or use of protocols, or a mixture of both. The results of this review indicate that pharmacists and nurses can effectively provide disease management and/or health promotion for older people with chronic disease in primary care. While there were improvements in patient outcomes no reduction in health service use was evident.

**Conclusion:**

When implementing skill mix changes such as task substitution it is important that the health professionals' roles are complementary otherwise they may simply duplicate the task performed by other health professionals. This has implications for the way in which multidisciplinary teams are organised in initiatives such as the GP Super Clinics.

## Background

In Australia, workforce shortages are occurring across a range of health disciplines, including general practitioners and practice nurses. In 2005, it was estimated that there was a shortfall of 800 to 1,300 GPs; 4% to 6% of the workforce. The nursing shortage was estimated at being between 10,000 and 12,000 nurses; addressing this would require doubling the number of nursing graduates [1]. The shortages are more acute in rural and remote Australia, particularly of doctors [2] and allied health professionals [3]. In addition, the workforce shortages are often accompanied by high staff turnover and poor distribution. Most sectors of the healthcare workforce are affected but it is particularly acute in primary care [4,5]. These shortages are occurring against a background of an ageing population with an increasing prevalence of chronic disease and higher consumer expectation [5].

Several responses have been proposed. Some are short-term fixes such as increasing the number of overseas-trained health professionals. Longer-term solutions include increasing the numbers and improving the distribution of graduates, and changing the way existing health professionals work, through multidisciplinary teams or changed skill mix. Since 2000, ten new medical schools and more medical training places have been established in an attempt to address medical workforce shortages and an increase in the number of nursing places. The aim of some of the new medical schools is to address local workforce need [6]; this is further reinforced by Bonded Scholarships, which tie the recipient to an area of workforce shortage such as rural and remote communities. It will take at least 10 years with current training pathways before these students finish their studies and join the medical workforce. In the meantime improved skill mix can be achieved through role enhancement, substitution, delegation or innovation in the use of existing health professionals to address workforce shortage. In this context, substitution is defined as expanding the breadth of a job by working across professional divides [7].

A systematic review of GP nurse substitution in primary care found that, in activities such as providing ongoing or first contact care, nurses achieved the same outcomes as GPs [8]. In some studies nurse care was felt to be superior to that provided by the GP as they spent more time with the patient and provided them with more information. There have been conflicting assessments of the cost-effectiveness of providing equivalent care from GPs or nurses (including nurse practitioners), although current evidence suggests that nurses and nurse practitioners deliver care less expensively in salary costs than GPs [9], but may use more health service resources [10]. The use of nurse practitioners does not always reduce GP workload; to avoid duplication careful consideration must be given to system redesign [11].

There is good evidence from systematic reviews that multidisciplinary care teams improve process of care and patient outcomes for patients with a range of chronic diseases [12-18]. To support effective multidisciplinary team interventions and to facilitate patient review and recall it is important to have a clear division of labour and clinical information systems [16]. In Australia, there has been some resistance to changing health professionals' roles to take on more of the work traditionally performed by doctors [19]. Another barrier is that Medicare rebates are largely limited to services provided by doctors. An item number for practice nurses to provide some chronic disease care on behalf of GPs was introduced in 2007; however, it remains difficult for practices to make best use of other providers such as practice nurses or pharmacists and to be reimbursed for providing these services. Increasing focus on integrated primary health care through initiatives such as GP Super Clinics [20] and Health One Centres in New South Wales [21] promises greater use of non-medical providers to deliver primary care. Formalising this process within the health system is important, as it provides a framework for scope of practice and quality assurance. This is particularly important in rural or remote areas where there may be a rapid turnover of staff and access to GPs is limited. This should be based on the best available local and international evidence [12-18].

The review explored the evidence for the effectiveness of skill mix changes in the form of task substitution between GPs and nurses or pharmacists in the care of older people living in the community. Older people are an expanding group with high prevalence of chronic disease; providing sufficient and appropriate care is becoming more challenging in the context of workforce shortages.

## Methods

Studies meeting the inclusion criteria for the review were identified by searching Medline, Embase, CINAHL, Cochrane Library (Issue 4, 2006), the Database of Abstracts of Reviews of Evidence (DARE) and the Joanna Briggs Institute (JBI) Library from 1990 to February 2007. Terms for skill mix developed by Sibbald and colleagues [7,8] were combined with terms for primary and community care developed during a previous review [12]. The Cochrane Effective Practice and Organisation of Care (EPOC) quality filter [22] was applied to include randomised controlled trials (RCTs), controlled clinical trials (CCTs), controlled before and after (CBA) and interrupted time series (ITS) studies for the detailed search strategy. Systematic reviews identified in the process were read and all papers that met the criteria for this review were added to the list of papers. The bibliographies of all experimental papers included were searched to identify additional studies.

Studies were included in the review if they were published in English during or after 1990 and if they addressed male or female adults aged 65 years and over living in the community. Older people living in hostels or nursing homes were excluded because their care arrangements are different. The interventions were defined as those that involved the planning and delivery of continuous care by nurses (this included practice nurses, community nurses and nurse practitioners), or pharmacists, compared to GPs. Studies were included if they met the study design criteria detailed as described in the EPOC methods, but papers were not excluded on the basis of quality because of the nature of many of the studies identified in this area; these were summarised. Experimental studies were included if they objectively measured health service use, quality of care or patient outcomes in a clinical setting, or self-report measures of known validity and reliability. Quality of care included process outcomes such as adherence to disease-specific guidelines, disease-specific measurements such as blood pressure, blood glucose, spirometry, weight, referrals and follow-up. Patient outcomes included disease control, self-report measures with known validity and reliability such as wellbeing, quality of life and disability scores. Patients' health service use, patient satisfaction, provider satisfaction and economic measures were also included. All papers underwent screening, verification and quality assessment by two reviewers before data extraction.

The skill mix interventions with evidence were categorised into two groups:

• Pharmacists substituting for GPs

• Nurses (practice nurses, community nurses and nurse practitioners) substituting for GPs.

Within each of these groups the interventions delivered by health professionals were categorised into three groups. Two reviewers (IH and SD) independently reviewed the intervention description and categorised the interventions as health promotion activities, disease management activities, such as self-management support and guideline-based care or both. A vote- counting method of analysis was used because the variety of studies did not permit use of meta-analysis [23]. For each of the outcome measures listed, if one of the recorded outcome measures showed a statistically significant improvement (p value < 0.05) it was coded as a statistically significant improvement. The number of papers reporting the outcome of interest was calculated as was the proportion of those that were significantly improved as a result of the intervention.

## Results

The search strategy identified 15,148 papers; after screening, verification and quality assessment data were extracted from 46 papers with a mean quality score of 9.9 (SD 2.08) and median score 10.0 (range 5-15). The majority were reports of RCTs; almost half targeted people with cardiovascular disease or diabetes (see Table 1). The health professionals and types of skill mix interventions are described in Table 2. The substitution of doctors by nurses was effective in disease management and health promotion for older people with chronic disease but the vote-counting method of analysis does not permit an estimate of the effect size (see Table 3). There was evidence that physiological outcomes of disease, adherence to disease management guidelines and treatment were improved with pharmacists or nurses compared to doctors. There was more evidence of effect for pharmacists than for nurses substituting for doctors. Patients were more likely to comply with their treatment regimes when managed by a pharmacist compared to a GP. There was no reduction in health service use when nurses or pharmacists substituted for doctors. Interventions that were most likely to be substituted were disease management according to published guidelines or a health promotion activity. Nurses and pharmacists received additional training specific to the study in order to provide the intervention. Training for the nurses was frequently provided by the GP, specialists or allied health providers such as diabetes educators. Pharmacist training included screening and behaviour change counselling and further postgraduate training for those studies where pharmacists had prescribing privileges.

**Table 1:** Characteristics of the primary research papers included in the review

**Study characteristics**	**Number (n = 46)**	**Percentage**
Study design		
Randomised controlled trial	30	65.2
Before and after (no control)	7	15.2
Controlled before & after	6	13.0
Controlled clinical trial	3	6.5
Year published		
Pre-2000	7	15.2
2000 and later	39	84.8
Settings		
Primary Care	35	76.1
Pharmacy	6	13.0
Community	1	2.2
Community based care	2	4.3
Managed Care Organisation	2	4.3
Intervention area		
Urban	30	65.2
Urban and rural	8	17.4
Rural	5	10.9
Remote	1	2.2
Not clear	2	4.3
Country		
USA	23	50.0
UK	12	26.1
Netherlands	4	8.7
Australia	2	4.3
Canada	2	4.3
Other	3	6.5
Chronic Disease		
Cardiovascular conditions	12	26.1
Diabetes & related conditions	12	26.1
Not mentioned	10	21.7
2 or more chronic conditions	6	13.0
Musculo-skeletal conditions	2	4.3
Mental conditions	3	6.5
Respiratory conditions	1	2.2

**Table 2:** Number and type of skill mix interventions by nurses and pharmacists substituting for GP

**Health Professional**	**Disease management role only**	**Health promotion role only**	**Both DM and HP**
Nurses	11	5	5
Pharmacist	14	3	8

**Table 3:** A summary of the effectiveness of task substitution by patient condition

	**Outcome Measures**							
**Medical conditions of patients**	**Professional adherence to guideline**	**Patient adherence to treatment**	**Patient service use**	**Patient physio-logical measure of disease**	**Patient quality of life**	**Patient health status**	**Patient Satisfaction**	**Patient functional status**
**Dr task substitution by nurse**								
All conditions	8 (10)	0 (1)	2 (12)	6 (9)	3 (8)	2 (8)	3 (8)	0 (2)
Respiratory			0 (1)		0 (1)		0 (1)	
Diabetes	5 (5)		0 (1)	3 (5)	0 (2)	1 (2)	0 (2)	0 (1)
Cardiovascular		0 (1)	1 (3)	1 (2)	3 (4)	1 (4)	0 (1)	0 (1)
Musculoskeletal					0 (1)			
Mental condition			0 (1)			0 (1)		
2 or more chronic condition	0 (2)		0 (1)	2 (2)				
Others								
Not specified	3 (4)		1 (5)			0 (1)	3 (4)	
**Dr task substitution by pharmacist**								
All conditions	6 (6)	8 (11)	2 (11)	13 (14)	3 (9)	4 (5)	5 (6)	
Respiratory					1 (2)		1 (2)	
Diabetes	3 (3)	1 (1)	0 (1)	6 (6)				
Cardiovascular		2 (2)	0 (2)	4 (5)	2 (3)	2 (2)	1 (1)	
Musculoskeletal						1 (1)		
Mental condition	1 (1)	1 (1)	1 (1)			1 (2)	1 (1)	
2 or more chronic condition	1 (1)	0 (2)	1 (2)	3 (3)	0 (2)		1 (1)	
Others								
Not specified	1 (1)	4 (5)	0 (5)		0 (2)		1 (1)	

Note1: Number in cells is the number of studies showing at least one significant outcome for that particular outcome measure

Note2: Number in bracket is the number of studies reporting at least one outcome measure in that particular category

## Discussion

With the recent change of government in Australia there has been a policy move towards multidisciplinary group general practice GP Super Clinics. In the current policy environment the results of this review provide health service providers, managers and policy-makers with evidence to support the role of the multidisciplinary team and task substitution in the management of people with chronic disease in primary care. It provides evidence that nurses or pharmacists can provide ongoing care or health promotion for older people, many of whom have chronic conditions as effective, or more effective, than that provided by a GP. Many of the interventions reviewed involved the use of disease management or health promotion guidelines.

While there were improvements in both the process of care and patient level outcomes, task substitution did not result in reduced use of health services. This may be because the nurses or pharmacists identified additional problems and made referrals to other health professionals or back to the GP. This is consistent with findings in an evaluation of community matrons working as case managers for frail elderly patients in the community in the UK [24] and a review of the impact of nurse practitioners on GP workload [11]. It highlights the importance of system redesign and the role of management to support an effective skill mix that ensures the task complements rather than duplicates the work of the GP.

The types of task substitution in this review were focused interventions to provide disease management and/or health promotion advice for a range of long-term conditions in the primary care setting. For nurses, disease management interventions included case management using guidelines, proactive follow-up, care planning, and goal setting. For pharmacists, the successful roles included medication review, patient management using algorithms, which sometimes included change of medication or dose adjustment medication compliance checks, risk factor screening, and counselling. Both nurses and pharmacists were involved in activities such as supporting patient self-management. All health professionals received additional training to undertake the task substitution.

There are some limitations to these findings. The vote-counting method of analysis does not allow a judgement about effect size associated with task substitution. The literature included in the review did not describe the length of appointment (or intervention) so it is difficult to judge the cost-effectiveness of such task substitution interventions.

### Policy implications

So what do these results mean for the delivery of primary care to older community-dwelling Australians with chronic disease? We know that well-trained nurses or pharmacists can provide health promotion or disease management according to guidelines that is equivalent to or better than that provided by a GP. In a climate of workforce shortages this review provides some evidence to support skill mix changes between health professionals, which may not currently be occurring in Australia and which may ease problems associated with workforce shortage in some areas. However, in Australia there has been a reluctance to embrace skill mix [19]. Duckett has argued for some time that the roles of some health professionals will need to change to address workforce shortages in Australia [25] but that in order for this to occur there must be a shift towards a more flexible workforce and inter-professional education. The current Medicare rebate system favours a medical model; until this becomes more flexible there is little incentive for change [26]. Duckett proposed an increase in the number of services other health professionals could provide on behalf of a GP as a means for providing some incentive for change. This has been effective in the UK where the practice incentives received in the Quality and Outcomes Framework were achieved partly through the work of practice nurses [27,28]. The payments are made at the practice level and it is for individual practices to decide who in the practice team will deliver this care [29]. In addition to a change in the fee structure there would need to be agreement on scope of practice and training of health professionals to ensure that those health professionals taking on additional tasks are doing so within an agreed regulatory framework, and are equipped to do so. To avoid duplication, at a local level it would be necessary for health professionals to agree on the tasks for substitution [11]. It would also be necessary to address mistrust and the differing agendas of health professionals and professional organisations. This might be achieved by a greater understanding of the roles and attitudes to multidisciplinary team-work that could be achieved through interdisciplinary education [30], which would be reinforced in practice if incentives supporting multidisciplinary team-work were comprehensive.

Current health policy in primary care is focused on the development of GP Super Clinics, such as Health One in New South Wales or South Australia's HealthPlus. These centres will house a range of health professionals working together, and provide opportunities for task substitution between health professionals, although they will not address payment mechanisms to support this. The Chronic Care Model [31] provides a framework for the way in which care for people with chronic diseases can be organised in primary care. There are six elements to the model: health care organisation; community resources; self-management support; delivery system design; decision support; and clinical information systems. There is good evidence of effectiveness for several elements of the Chronic Care Model in primary care in the context of the Australian health care system [12,13]. Creating well-trained practice teams with a clear or flexible division of labour is an essential step in this process, which fits well with the Super Clinics model of care [32]. Bodenheimer described several case studies of workforce redesign to support people with chronic disease with proactive follow-up for these patients. Underpinning this team approach to care are disease registers and shared notes that have been shown to improve patient level outcomes [32] and will need to be incorporated into the structure of GP Super Clinics if multidisciplinary team care is to be effective.

High quality and comprehensive practice nurse training is needed to support such extended roles. A number of authors have identified that, while accredited courses for practice nurses exist in Australia, the current practice environment provides nurses with little incentive to undertake additional training unless their role will develop to include the new skills [33-35]. GPs have little incentive to pay for nurse training if there are no item numbers to support the extended practice nurse roles. Even in the UK, where practice nursing is more developed, a recent survey identified that 20% of nurses with an advanced respiratory care role had not undertaken accredited training [36]. There are similar findings from a survey of nurses involved in chronic disease care in Australia [37]. The challenge for health professionals and policy-makers in Australia is to provide an environment which values highly trained nurses in general practice and provides opportunities for them to utilise their skills to complement the work of GPs in providing high quality chronic disease management; only then will more nurses and GPs be prepared to invest in training.

At a practice level there is increasing acceptance of the role and value of skill mix. In rural and regional areas the utilisation and acceptance appears more advanced, probably on the basis of necessity [38-41]. These innovations in skill mix have occurred in the context of a service vacuum, and have been encouraged as they have not encroached on the work of other health professionals and have been negotiated locally. A recent systematic review by Greenhalgh et al. [42] explored the mechanisms involved in the diffusion of innovation which may be helpful to consider here. Large organisations with semi-autonomous units and specialist knowledge support the spread of innovation. Primary care is an example of a large overarching health system with general practices as the semi-autonomous units. A policy push can facilitate change while enabling modification of the interventions at a local level [12,13]. The policy push for Australian primary care would be the removal of funding barriers that currently prevent practices from making better use of other health professionals such as practice nurses and pharmacists. The GP Super Clinics provide an ideal opportunity to bring the multidisciplinary team together, but they are unlikely to succeed unless different team members' skills can be utilised and reimbursed. The Super Clinics will provide the structure to negotiate the scope of practice to avoid duplication.

Most of the discussion has focused on the role of the practice nurse, but this review also demonstrated that pharmacists were effective at providing health promotion and disease management interventions, and some of these interventions involved the ability to prescribe or modify prescriptions. The current model of community pharmacy with little direct communication with general practice needs revisiting to more effectively take account of the potential skill set of pharmacists.

## Conclusion

Task substitution between GPs and practice nurse or pharmacists is effective in improving the quality of care for older Australians living in the community. Involving practice nurses or pharmacists in the provision of disease management and health promotion may make some contribution to GP shortages. While task substitution improves quality of care it may not reduce costs. The challenge for Australia is how to create an environment that supports workforce development and uses skill mix and team work to address workforce shortage.

## Competing interests

The authors declare that they have no competing interests.

## Authors' contributions

SD participated in the design of the review, carried out the database searches, conducted the narrative synthesis and drafted the manuscript. JM, DP, NZ and BS participated in the design of the review, conceptual framework and contributed to the narrative synthesis. IH participated in the design of the review, carried out the data extraction and narrative synthesis. All authors read and approved the final manuscript.
